# Perception, Practices Towards Research and Predictors of Research Career Among UG Medical Students from Coastal South India: A Cross-Sectional Study

**DOI:** 10.4103/0970-0218.58388

**Published:** 2009-10

**Authors:** HN Harsha Kumar, S Jayaram, Ganesh S Kumar, J Vinita, S Rohit, M Satish, K Shusruth, Nitin, Akhilesh

**Affiliations:** Department of Community Medicine, Mangalore-575 001, Karnataka, India; 1 Department of Community Medicine, Kasturba Medical College, Mangalore-575 001, Karnataka, India

**Keywords:** Cross-sectional, medical students, research

## Abstract

**Background:**

The number of physician scientists worldwide is decreasing. A review of literature suggests paucity of information examining perceptions and practices towards research among medical undergraduate students in India. Hence, this study was undertaken.

**Objectives:**

To understand (a) the awareness, skills, perceptions and practices among undergraduate (UG) medical students towards research, (b) the factors responsible for willingness to take up research as a career among the undergraduates.

**Material and Methods:**

This is a questionnaire-based qualitative study. This study was conducted in Kasturba Medical College, Mangalore. A pre-tested questionnaire examining their awareness, perceptions and practices towards research in medical field was used. Consent was obtained from the Dean of the College and student participation was voluntary.

**Analysis:**

The information was analyzed using SPSS version 11. Univariate and Multivariate analyses were done to know the willingness to consider research as a career.

**Results:**

A total of 471 students responded giving a response rate of 55.41%. Nearly 70% were aware about research though their level of awareness varied. Various skills of conducting research were known to 47% of the students. Most (76%) were part of a research team mainly as a part of the medical curriculum, a few (8.3%) were confident of research as a career option. The multivariate reveals that those with good skill and students who involved in research in addition to curriculum were more likely to take up research as career option/would continue to do research in future.

**Conclusions:**

Good training and student support programs exclusively for research would motivate students to opt for research careers.

## Introduction

There has been concern in recent years about a decline in the number of new physician-scientists worldwide. Many factors, such as increasing cost of education, higher financial returns from clinical careers, reduction of research budgets with increased competition for research funding, may have contributed to this decline.(1) Advances in bio-medical research during the last decade have highlighted the necessity of attracting greater numbers of physicians to careers that include a research component. Physician participation in research is essential to increase the number of clinical and research studies performed.(2) Poor training in research skills in medical curriculum is thought to be responsible for this.(3) The review of literature suggests that in India, there is paucity of information examining the awareness, perceptions and practices of medical students towards research.(4) Apart from knowing the perceptions and practices, it is also important to find out the factors which determine the medical student's choice of research as a career. Hence, this study was undertaken.

## Materials and Methods

The study was conducted in Kasturba Medical College, Mangalore. There are four batches with 250 students each, making a total of 1000 in the college. The students attend the Community Medicine postings in the third, fourth and seventh semesters. As part of their curriculum, the students are supposed to complete a “Students Research Project” during their second posting to our department. Some students undertake research in addition to their curriculum like ICMR (Indian Council of Medical Research) student projects, projects going on in various departments of the college.

This is a questionnaire-based qualitative study. This study was conducted from January 1 to February 3, 2007. All the medical undergraduate students of the college were considered for the study while participation was voluntary.

A questionnaire was devised to collect the following three components of information from the students: 1. Information to know their awareness, and perceptions about importance of research in medical field. 2. Questions to assess their practices i.e. attempts to write, conduct projects and publish in journals. 3. Opinion regarding choosing research as their career. This questionnaire had a mix of open ended, closed ended single and multiple response questions. This was pre-tested on some students. Certain modifications had to be made like, rephrasing some questions, making some questions open ended and permitting multiple responses to some questions etc.) to suit our purpose. A paragraph explaining the purpose of the study, seeking their consent and requesting their participation in the study was added at the beginning of the questionnaire. It was also made clear that the participation in the study was voluntary. The same was also announced in the class before distribution of the questionnaire.

Permission was obtained from the Dean of the College to conduct the study. The students were approached in the class rooms and the purpose of visit explained. As the students did not have to mention their names or registration numbers, the confidentiality of their responses was assured. Questionnaires were distributed and after 30 minutes the questionnaires were collected.

If some students did not want participate in the study, the reasons were to be mentioned in the beginning of the questionnaire.

### Data analysis

Data was entered in SPSS version 10 and the results were analyzed. Results have been expressed in proportions. Some of the responses to open ended questions were recoded and analyzed. Analysis was done to know the factors that would motivate undergraduate medical students to take up research as a career. Univariate analysis was done to know the unadjusted Odds Ratios reflecting the likelihood of taking up research as a career. To remove the effect of confounding and assess the independent effects of the study variables (ie, Gender, Skill, Familiarity with research, Participation in research work with in curriculum and in addition to curriculum) on the dependent variable (ie, Taking up Research as a career option), Multiple logistic regression was performed. This gave us the adjusted Odds ratios with its 95% Confidence Intervals.

## Results

As one of the batches (ninth semester) was about to appear for exams, most of the students of this batch could not be found in the class room at the time of data collection. A total of 850 students could be found in class rooms of which 471 responded giving a response rate of 55.41%. Of these, 196 were from the first year (Joined in 2007), 176 form the second year (Joined in 2006) and 99 from the final year (two batches ie, Part I and Part II joined in 2005 and 2004 respectively). The proportion of males and females was nearly equal (51.2% and 48.8% respectively). A large percentage of students (61.3%) who did not respond, did not give any specific reason. Some (22.4%) considered it a waste of time / not worth while participating, some others (10.6%) said they did not have interest in research/would like a different topic. The remaining students (5.7%) answered very few questions, necessitating their exclusion from analysis.

Awareness, perceptions and practices of the students reflected in their responses to the questions is presented in Table 1. Some of the questions were not relevant for first years as the teaching of research methodology starts in the second year (third semester). Some open ended questions like, which type of internet searching, for research, did the students resort to (multiple responses permitted), evoked following responses: Google searching (74%), Yahoo (56%), Wikipedia (43%), Pubmed (18%). Only 16% of students had made an attempt to publish like, submitting the article to journal or their articles were rejected. Four students had managed to publish in indexed peer reviewed journals. Students cited more than one reason for their not being involved in research in addition to curriculum like ‘shortage of time’ (52.1%), lack of financial/academic benefit (56.3%), not interested to undertake research as a career (32.4%), ‘want to be a clinician only (37.8%), ‘doctor should work in hospital (24.3%),’ no idea about research career (29.6%).

**Table 1:** Research in medical field: Awareness, perceptions, practices of UG medical students

Questions exploring their awareness, perceptions and practices	I year students (n=196) (%)	II year students (n=176) (%)	Final year students (n=99) (%)
Do you think research in medical field is important?			
Yes	30 (15.3)	56 (31.8)	91(91.9)
No	40 (20.4)	34 (19.3)	5 (5.1)
Don't know	126 (64.3)	86 (48.9)	3 (3)
Is it important for medical students to know about research methodology?			
Yes	28 (14.3)	89 (50.6)	80 (80.8)
No	168 (85.7)	87 (49.4)	19 (19.2)
What do you think of research career for a doctor?			
Good	8 (4.1)	12 (6.8)	9 (9.1)
Financially bad option	12 (6.1)	25 (14.2)	21(21.2)
No status/Respect	20 (10.2)	31 (17.6)	28 (28.3)
Not good	16 (8.2)	27 (15.3)	30 (30.3)
Don't know	140 (71.4)	81 (46)	11 (11.1)
Have you ever been part of a research team?*			
Yes	-	110 (62.5)	99 (100)
No		66 (37.5)	-
Have you been a part of research team in addition to your curicurrulum?			
Yes	3 (1.5)	31(17.6)	13 (13.1)
No	193 (98.5)	145 (82.4)	86 (86.9)
What are the sources of information for doing research?			
Books	103 (52.6)	34 (19.3)	41 (41.4)
Equipment	76 (38.8)	57 (32.4)	21 (21.2)
Journals	8 (4.1)	97 (55.1)	87 (87.9)
Library	96 (49)	46 (26.1)	6 (6.1)
Internet	56 (28.6)	102 (58)	92 (92.2)
Don't know	41 (20.9)	4 (2.3)	0 (0)
Are you familiar with writing of protocol?*			
Yes	-	42 (23.9)	87 (87.9)
No		134 (76.1)	12 (12.1)
Did you make an attempt to publish?*			
Yes	-	29 (16.5)	34 (34.3)
No		147 (83.5)	65 (65.7)
Would you take up research as your career option?		
Yes	2 (1)	15 (8.5)	15 (15.2)
No	194 (99)	161 (91.5)	84 (84.8)

* Question not applicable to first year students.

The results of univariate and multivariate analyses are presented in Table 2. On multivariate analysis only two factors ie, ‘presence of good skill’ and having ‘participated in research in addition to curriculum’ came out to be significant, reflecting the affect of confounding.

**Table 2:** Determinants of willingness to opt for research career among medical students - Results of univariate and multivariate analysis

Determinant/Factors	Univariate - Unadjusted odds ratio (OR) with 95% CI		Multivariate - Adjusted odds ratio (OR) with 95% CI		*P* value
			
	OR	(95% CI)	OR	(95% CI)	
Sex					
Male	2.767	1.12 - 5.17	1.262	0.55 - 2.91	0.58
female	-		-		
Familiarity					
Yes	3.01	1.56 - 5.64	1.61	0.70 - 2.66	0.16
No	-		-		
Skill					
Good	4.86	3.14 - 9.98	2.18	1.76 - 7.36	0.04*
Inadequate	-		-		
Participation in research work with in curriculum	4.22	2.25 - 7.90	1.57	0.47 - 5.27	0.24
Yes	-		-		
No					
Research in addition to curriculum					
Yes	12.23	8.24 - 36.03	7.81	5.14 - 15.42	<0.001*
No	-		-		

* *P* of <0.05 was considered to be significant.

## Discussion

The response rate decreased from the first to final year as final year students stated that they do not want to spare time for this purpose. The proportion of males and females were roughly equal. There were no significant gender differences in preferences for a research career. Male preponderance for research career has been reported by Guelich *et al*.(5) They used a cohort study design to ascertain the gender differences by tracking the careers of the doctors. From our study we cannot rule out the possibility of gender differences in their actual career choices in future. The training of the undergraduates in research methodology with in the curriculum has improved their awareness and their perceptions of importance of research methodology [Table 1]. A good training is known to improve the awareness and skill of medical students and help them develop a positive attitude towards research.(6,7) Though the student's awareness increased because of participation in research and teaching as a part of curriculum, this by itself is not an important determinant of their research career choice [Table 2]. Many students felt that research as a career choice was neither ‘financially rewarding’ nor had that ‘status’ [Table 1]. It has been found that good financial support systems and exclusive support programs for research could increase chances of students taking up research as their career choice or make sure they associated with research irrespective of their future careers.(8,2) This underlies the need to have such support systems to promote the more doctors from taking up research careers or be associated with research irrespective of their specialties. Students who attempted research in addition to their curriculum had or were currently participating in Indian Council of Medical Research (ICMR) - STS (Short Term Studentship). Such students who pursued research, in addition to the curriculum, have the willingness to consider research as career [Table 2]. An intensive training or exposure of UG medical students to research will increase their chances of pursuing a research career.(9,2)

A few students had the willingness to consider research as their career. However, lack of proper support systems (except for ICMR STS projects) in our country, and lack of clarity of ‘research path’ (unlike a clinical branch) seem to demotivate the students from a research oriented career, though they have the awareness and skills.

Apart from the higher non-response rates among final year students, the response bias of students who participated, might have affected the results to some extent. There might be differences between their stated career intentions and their final career choices which can be found out form a cohort study. Non-response rates might have limited the utility of logistic regression though it does give us an idea of the factors which play a role in the willingness of the students to take up research as a career.

The reasons quoted by students, like ‘lack of any financial/academic benefit’, ‘no status/respect’, ‘no idea of research career’ emphazise the need to have student support systems to enhance the number of physician scientists in the long run.
